# Effects of combination therapy using antithrombin and thrombomodulin for sepsis-associated disseminated intravascular coagulation

**DOI:** 10.1186/s13613-017-0332-z

**Published:** 2017-11-02

**Authors:** Toshiaki Iba, Akiyoshi Hagiwara, Daizoh Saitoh, Hideaki Anan, Yutaka Ueki, Koichi Sato, Satoshi Gando

**Affiliations:** 1 Department of Emergency and Disaster Medicine, Juntendo University Graduate School of Medicine, 2-1-1 Hongo Bunkyo-ku, Tokyo, 113-8421 Japan; 2 National Center for Global Health and Medicine, Emergency Medicine and Critical Care, Tokyo, Japan; 3 Division of Traumatology, Research Institute, National Defense Medical College, Tokyo, Japan; 4 Emergency Medical Center, Fujisawa City Hospital, Fujisawa, Japan; 5 Department of Acute Critical Care and Disaster Medicine, Tokyo Medical and Dental University, Tokyo, Japan; 6 Department of Surgery, Juntendo Shizuoka Hospital, Juntendo University Graduate School of Medicine, Izunokuni-shi, Japan; 7 Division of Acute and Critical Care Medicine, Department of Anesthesiology and Critical Care Medicine, Hokkaido University Graduate School of Medicine, Sapporo, Japan

**Keywords:** Disseminated intravascular coagulation, Sepsis, Antithrombin, Thrombomodulin, Propensity analysis

## Abstract

**Background:**

No single anticoagulant has been proven effective for sepsis-associated disseminated intravascular coagulation (DIC). Thus, the concomitant use of antithrombin concentrate and recombinant thrombomodulin has been conceived. This observational study was conducted to investigate the efficacy and safety of this combination therapy.

**Methods:**

A total of 510 septic DIC patients who received antithrombin substitution were retrospectively analyzed. Among them, 228 were treated with antithrombin and recombinant thrombomodulin (combination therapy) and the rest were treated with antithrombin alone (monotherapy). Propensity score matching created 129 matched pairs, and 28-day all-cause mortality, DIC scores, the sequential organ failure assessment (SOFA) scores, and the incidence of bleeding were compared.

**Results:**

A log-rank test revealed a significant association between combination therapy and a lower 28-day mortality rate (hazard ratio 0.49, 95% confidence interval 0.29–0.82, *P* = 0.006) in the matched pairs. The DIC scores and the SOFA scores in the combination therapy group were significantly lower than those in the monotherapy group on Day 4 and Day 7. The incidence of bleeding did not differ between the groups (2.11 vs. 2.31%, *P* = 1.000).

**Conclusions:**

The current study demonstrated the potential benefit of adding recombinant thrombomodulin to antithrombin. The co-administration of these two anticoagulants was associated with reduced mortality among patients with sepsis-induced DIC without increasing the risk of bleeding.

**Electronic supplementary material:**

The online version of this article (10.1186/s13613-017-0332-z) contains supplementary material, which is available to authorized users.

## Background

Anticoagulant therapy for sepsis-associated disseminated intravascular coagulation (DIC) is widely performed in Japan [1], and antithrombin concentrate and recombinant thrombomodulin are the two most popular agents utilized for this treatment [2]. However, not a single anticoagulant has proven to be effective. Furthermore, neither of the above-mentioned agents has been recommended for use outside Japan [3, 4]. To examine the effects of recombinant thrombomodulin, Hayakawa et al. [5] conducted a retrospective multicenter survey examining 1784 sepsis-associated DIC cases. They created 452 propensity score-matched pairs and performed a logistic regression analysis. As a result, a significant association between recombinant thrombomodulin use and lower mortality (odds ratio [OR] 0.757; 95% confidence interval [CI] 0.574–0.999, *P* = 0.049) was recognized. The same group also performed a similar analysis on antithrombin concentrate and reported that the inverse probability of a treatment-weighted propensity score analysis indicated a statistically significant association between antithrombin supplementation and lower mortality (OR 0.748, 95% CI 0.572–0.978, *P* = 0.034). However, a propensity score-matched analysis did not show a significant association in a latter analysis [6]. In contrast to the situation in Japan, the international guidelines for sepsis do not recommend the use of antithrombin, and recombinant thrombomodulin is still not available outside Japan [7]. Despite the lack of robust evidence, the concomitant use of antithrombin and recombinant thrombomodulin has become popular in clinics, and recent post-marketing surveys have reported that combination therapy is now used in 50% of cases, at present [8]. Regarding the efficacy of combination therapy, available information remains sparse and the results are inconsistent. We formerly performed a logistic regression analysis among septic DIC patients who had undergone antithrombin supplementation and reported that the co-administration of recombinant thrombomodulin was a significant factor affecting survival [9]. Since the number of patients who received combination therapy was relatively small in that study, we repeated the survey and accumulated 159 patients in the second study [10]. This second survey demonstrated that the 28-day survival outcome in the combination therapy group was 80.5%, while it was only 63.9% in the antithrombin monotherapy group; this difference was statistically significant. Regarding the bleeding incidence, combination therapy is reportedly not associated with a risk of bleeding [10]. Since information regarding the effects and adverse effects of combination therapy is still limited [11], we planned to examine these issues in the third survey.

## Methods

### Patient selection

This post-marketing surveillance was performed as a multi-institutional, post-marketing survey. A total of 570 sepsis-associated DIC patients with an antithrombin activity ≦70% who were treated between June 2014 and June 2016 were registered. For the diagnosis of DIC, the Japanese Association for Acute Medicine (JAAM)-DIC criteria (Additional file 1: Supplement Table 1) [12] were utilized. Patients with a history of an allergic shock reaction to antithrombin, with major bleeding, an age of younger than 18 years old, or who were pregnant were excluded.

### Ethics, consent and permissions and consent to publish

The survey was performed under the supervision of the Japanese Ministry of Health, Labour and Welfare (JMHW) and was conducted in accordance with the Declaration of Helsinki and Good Vigilance Practice and Good Post-marketing Study Practice. Since the complete anonymization of personal data was performed upon data collection, the ethical committee of Juntendo University waived the need to obtain informed consent and the patients’ agreement. In the same reason, the institutional committee judged that the consent to publish was not required.

### Treatment

When the patients met the JAAM-DIC criteria and had an antithrombin activity level of ≦ 70%, antithrombin concentrate (Nihon Pharmaceutical Co. Ltd, Tokyo, Japan) was administered for up to 3 consecutive days unless the patient died or treatment was stopped for any justifiable reason. The concomitant use of other anticoagulants was not prohibited, and recombinant thrombomodulin (TM-α; Asahi Kasei Parma Corporation, Tokyo, Japan) was administered intravenously according to the drug manufacturer’s recommendation (0.06 mg/kg/day for 6 days by either intravenous bolus injection or intravenous infusion over 15 min via a catheter). Standard sepsis care was performed, and platelet concentrate and fresh-frozen plasma were used as substitution therapy, if necessary [13].

### Data collection

The baseline data for the coagulation markers including fibrinogen/fibrin degradation products (FDP), D-dimer, prothrombin time (PT) ratio, platelet counts and antithrombin activity were measured before the treatment. Systemic inflammatory response syndrome (SIRS) score, sequential organ failure assessment (SOFA) score, and JAAM-DIC score were also calculated. Serial data for each coagulation marker, SIRS score, SOFA score and JAAM-DIC were also measured after the start of treatment (Day 2, Day 4, Day 7).

Survival was recorded until Day 28. The bleeding events were recorded throughout the observation period. Major bleeding was defined as bleeding that was either fatal, involved the failure of a critical organ, or was associated with a decrease in the hemoglobin level of 2.0 g/dL or more or required the infusion of 2 or more units of blood. The platelet count and other coagulation profiles were measured in local laboratories.

### Statistical analysis

Student’s *t* test, Mann–Whitney test, and Fisher’s exact test were used to compare covariates between patients who received antithrombin alone (monotherapy group) versus antithrombin and recombinant thrombomodulin (combination therapy group). Bonferroni’s correction was used to compare DIC score and SOFA score between two groups.

Cox’s proportional hazards model (Cox hazard) was applied to evaluate the effectiveness of combination therapy. We selected some possible confounding covariates from the baseline characteristics and calculated a variance inflation factor (VIF). Finally, we set age, sex, baseline SOFA score, baseline DIC score, and antithrombin activity at baseline as confounding covariates. Then, a propensity score matching (PSM) was performed with these covariates.

A caliper width of s propensity score matching was set 0.06. Using this caliper width, we performed one-to-one nearest-neighbor matching without replacement between two groups.

To evaluate an effect size in the two matched groups, we calculated the standardized difference for continuous data and phi coefficient for categorical data. Log-rank test was used to compare two survival curves between monotherapy group and combination group. Data are expressed as a number (%), mean ± standard deviation (SD), or median (interquartile range), as appropriate. For all the reported results, *P* < 0.05 or *P* < 0.017 (0.05/3, Bonferroni’s correction) was considered to denote statistical significance. R version 3.1.3 was used for all analysis, and SPSS 24.0 for Windows (IBM SPSS Inc., Chicago, IL) was validated for these analyses.

## Results

### Baseline characteristics

**Fig. 1:**
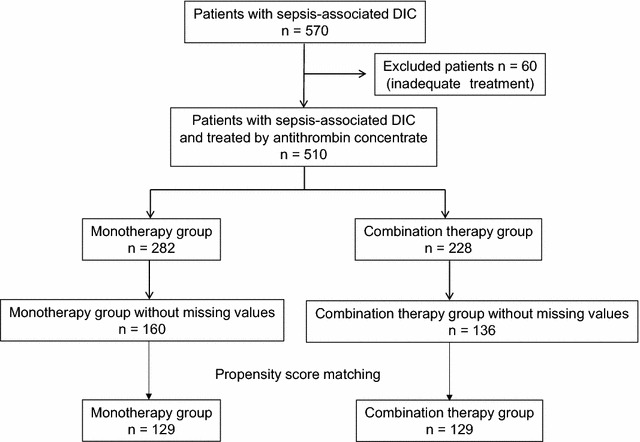
Patient selection for the evaluation of antithrombin concentrate and recombinant thrombomodulin combination therapy. *DIC* disseminated intravascular coagulation

A total of 570 patients were registered in this survey; however, 60 cases were excluded because their treatments did not meet the study’s criteria. Twenty-eight cases had an antithrombin activity > 70% when the treatment was initiated. In 16 cases, antithrombin activity was not measured. In the other 16 cases, antithrombin was not administered on the day of diagnosis. Data from 510 cases were used in the following analyses. Among them, 228 were treated with antithrombin and recombinant thrombomodulin (combination therapy group), and the remaining 282 were treated using antithrombin alone (monotherapy group). As for the infection focus, the respiratory system was the most frequent (29.6% 151/510). The baseline characteristics of the unmatched combination therapy and monotherapy groups are presented in Table 1. Propensity score matching created 129 matched pairs (Fig. 1). All the effect sizes of confounding covariates used by the propensity score were ≦ 0.1 for the matched patients, and the characteristics of the two groups were appropriately balanced (Table 2).

**Table 1:** The baseline characteristics of the enrolled patients (*n* = 510)

Factors	Monotherapy group *n* = 282	Combination therapy group *n* = 228	*P* value	Missing value
Survival at day 28 (%)				
No	81 (28.7)	51 (22.4)	0.127	0
Yes	201 (71.3)	177 (77.6)		
Age (mean [SD])	71.7 (14.7)	72.3 (15.6)	0.663	0
Sex (%)				
Female	115 (40.8)	88 (38.6)	0.682	0
Male	167 (59.2)	140 (61.4)		
Infection focus (*n*, %)				
Respiratory system	91 (32.3)	60 (26.3)	0.172	0
Gastrointestinal system	69 (24.5)	66 (28.9)	0.268	0
Biliary system	35 (12.4)	28 (12.3)	1.000	0
Urinary system	36 (12.8)	39 (17.1)	0.208	0
Musculoskeletal	17 (6.0)	7 (3.1)	0.142	0
Skin and soft tissue	10 (3.5)	7 (3.1)	0.809	0
Central nerve system	2 (0.7)	2 (0.9)	1.000	0
Other	13 (4.6)	15 (6.6)	0.337	0
Unknown	35 (12.4)	20 (8.8)	0.199	0
Surgical intervention	23 (8.2)	34 (14.9)	0.023	0
Non-surgical drainage^#^	5 (1.8)	10 (4.4)	0.113	0
Baseline SOFA score median [25, 75%]				
Total SOFA	10.0 [7.0, 13.0]	11.0 [8.0, 13.0]	0.062	120
Coagulation	2.0 [1.0, 3.0]	2.0 [1.0, 3.0]	0.402	15
Hepatic	0.0 [0.0, 2.0]	0.0 [0.0, 1.0]	0.020	33
Cardiovascular	2.0 [0.0, 4.0]	3.0 [0.3, 4.0]	0.001	19
CNS system	2.0 [0.0, 3.0]	2.0 [1.0, 3.0]	0.312	60
Renal system	1.0 [0.0, 2.0]	1.0 [0.0, 2.0]	0.140	21
Respiratory system	2.0 [1.0, 3.0]	2.0 [1.0, 3.0]	0.038	88
Baseline DIC score median [25, 75%]				
Total DIC score	5.0 [4.0, 6.0]	5.0 [4.0, 7.0]	0.039	29
SIRS score (*n*, %)				
0 point	130 (46.4)	77 (34.4)		
1 point	150 (53.6)	147 (65.6)	0.006	6
Platelet score	3.0 [1.0, 3.0]	3.0 [1.0, 3.0]	0.182	1
FDP score	3.0 [1.0, 3.0]	3.0 [1.0, 3.0]	0.242	14
PT ratio score (*n*, %)				
0 point	53 (19.9)	33 (14.5)		
1 point	214 (80.1)	195 (85.5)	0.123	15
Baseline laboratory score median (SD)				
Platelet count (× 10^9^/L)	99.5 (77.6)	89.6 (68.1)	0.130	1
FDP (μg/mL)	56.6 (150.3)	48.8 (66.7)	0.526	110
D-dimer (μg/mL)	26.8 (46.7)	19.7 (29.1)	0.053	121
PT ratio	1.97 (6.08)	1.55 (0.52)	0.298	15
Antithrombin activity (%)	49.6 (13.8)	47.3 (12.7)	0.058	17

The data were shown as mean (standard deviation; SD) or median [25th percentile, 75th percentile]

As the SIRS score and the PT ratio score were composed of binary data, Fisher’s exact test was performed

Non-surgical interventions are as follows: percutaneous transcatheter abscess drainage, urinary tract stenting, biliary tract stenting

*n* number, *SOFA* sequential organ failure assessment, *CNS* central nervous system, *DIC* disseminated intravascular coagulation, *SIRS* systemic inflammatory response syndrome, *FDP* fibrinogen/fibrin degradation products, *PT* prothrombin time

^#^ For the selection of covariates, a variance inflation factor (VIF) was calculated and the covariates with VIF ≧ 5 were excluded. Finally, age, sex, baseline SOFA score, baseline DIC score, and antithrombin activityused were selected.

**Table 2:** The baseline characteristics of the patients after propensity score matching (*n* = 258)

Factors	Monotherapy group *n* = 129	Combination therapy group *n* = 129	*P* value	Effect size
Age, mean (SD)	73.3 (12.0)	73.8 (11.5)	0.723	0.044^a^
Sex (*n*, %)				
Female	54 (41.9)	52 (40.3)	0.899	0.064^a^
Male	75 (58.1)	77 (59.7)		
Infection focus (*n*, %)				
Respiratory system	58 (45.0)	38 (29.5)	0.014	0.160
Gastrointestinal system	22 (17.1)	34 (26.4)	0.096	0.113
Biliary system	19 (14.7)	15 (11.6)	0.581	0.046
Urinary system	12 (9.3)	23 (17.8)	0.068	0.125
Musculoskeletal	8 (6.2)	4 (3.1)	0.376	0.074
Skin and soft tissue	5 (3.9)	3 (2.3)	0.722	0.045
Central nerve system	0	1 (0.8)	1.000	0.062
Others	3 (2.3)	8 (6.2)	0.216	0.096
Unknown	14 (10.9)	12 (9.3)	0.837	0.026
Surgical intervention	8 (6.2)	19 (14.7)	0.040	0.139
Non-surgical drainage^#^	1 (0.8)	4 (3.1)	0.370	0.084
Baseline SOFA score median [25, 75%]				
Total SOFA	10.7 [2.0, 22.0]	10.8 [3.0, 19.0]	0.849^a^	0.025^a^
Hepatic	1.0 [0.0, 3.0]	0.0 [0.0, 3.0]	0.015	0.182
Cardiovascular	3.0 [0.0, 4.0]	3.0 [0.0, 4.0]	0.266	0.151
CNS system	2.0 [0.0, 4.0]	2.0 [0.0, 4.0]	0.678	0.026
Renal system	1.0 [0.0, 4.0]	1.0 [0.0, 4.0]	0.656	0.028
Respiratory system	2.0 [0.0, 4.0]	2.0 [0.0, 4.0]	0.266	0.069
Baseline DIC score Median [25, 75%]				
Total DIC score	5.7 [2.0, 8.0]	5.7 [2.0, 8.0]	0.935^a^	0.013^a^
SIRS score (*n*, %)				
0 point	51 (39.5)	37 (28.7)		
1 point	78 (60.5)	92 (71.3)	0.088	0.114
Platelet score	3.0 [0.0, 3.0]	3.0 [0.0, 3.0]	0.778	0.048
FDP score	3.0 [0.0, 3.0]	3.0 [0.0, 3.0]	0.829	0.105
PT ratio score (*n*, %)				
0 point	15 (11.6)	21 (16.3)		
1 point	114 (88.4)	108 (83.7)	0.369	0.067
Baseline laboratory score mean (SD)				
Platelet count (× 10^9^/L)	8.60 (6.29)	8.47 (5.04)	0.853	0.023
FDP (μg/mL)	73.9 (191.1)	51.11 (73.35)	0.206	0.158
D-dimer (μg/mL)	28.3 (48.9)	21.1 (24.3)	0.166	0.185
PT ratio	2.42 (8.68)	1.50 (0.41)	0.232	0.150
Antithrombin activity (%)	45.6 (14.1)	47.7 (11.7)	0.928	0.011^a^

^#^ For the selection of covariates, a variance inflation factor (VIF) was calculated and the covariates with VIF ≧ 5 were excluded. Finally, age, sex, baseline SOFA score, baseline DIC score, and antithrombin activityused were selected.

^a^Confounding covariates used by the propensity score (age, sex, baseline SOFA score, baseline DIC score, and antithrombin activity)

Non-surgical interventions are as follows: percutaneous transcatheter abscess drainage, urinary tract stenting, biliary tract stenting

When the basic assumptions of Student’s *t* test were satisfied, data were shown mean (standard deviation) and the effect size was calculated using Cohen’s *d*. When the basic assumptions of Student’s *t* test were not satisfied, Mann–Whitney *U* test was performed and data were shown median [25 percentiles, 75 percentiles]. And the effect size was calculated using the following formula, *Z*-scores/a square root of sample number. For two-by-two contingency table, phi coefficient was used

*n* number, *SD* standard deviation, *SOFA* sequential organ failure assessment, *DIC* disseminated intravascular coagulation

### Effects on survival among the patients after propensity score matching

**Fig. 2:**
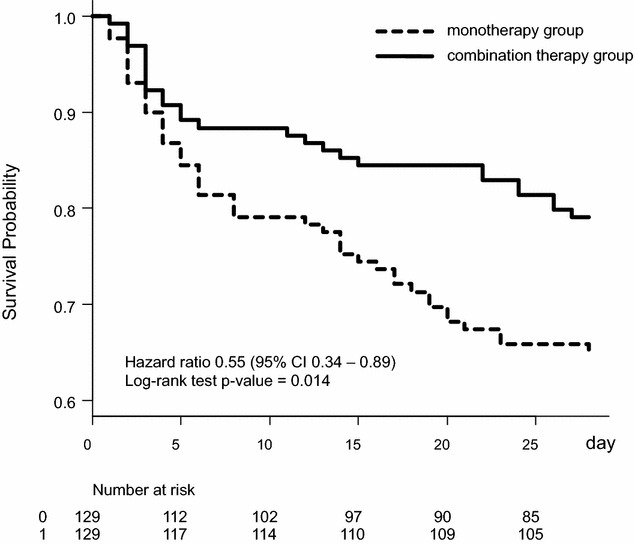
Survival plots for patients in the propensity score-matched combination therapy and monotherapy groups. The 28-day survival rate was significantly higher in the combination therapy group (79.8%) than in the monotherapy group (70.0%) (*P* = 0.014, log-rank test). Hazard ratio 0.55 (0.34–0.89).

The Kaplan–Meier survival curves for the two groups are shown in Fig. 2. The hazard ratios (HRs) for 28-day mortality for combination therapy were 0.62 (95% CI 0.40–0.98, *P* = 0.043 [Cox’s proportional hazards model) and 0.55 (95% CI 0.34–0.89, *P* = 0.014 [propensity score matching]), and significant associations were observed between the combination therapy and a lower 28-day mortality (Table 3).

**Table 3:** Hazards ratio analysis in patients treated with combination therapy

Case number	Model	Hazard ratio (95% CI)	*P* value
510	Unadjusted	0.71 (0.45–1.11)	0.131
296^a^	Cox hazard	0.62 (0.40–0.98)	0.043
258	PS matching	0.49 (0.29–0.82)	0.006^$^

*CI* confidence interval, *Cox hazard* Cox’s proportional hazards model, *PS* propensity score

^a^Complete case number without missing values

^$^ *P* values were calculated using a log-rank test

### Effects on coagulation markers, DIC score and SOFA score

**Fig. 3:**
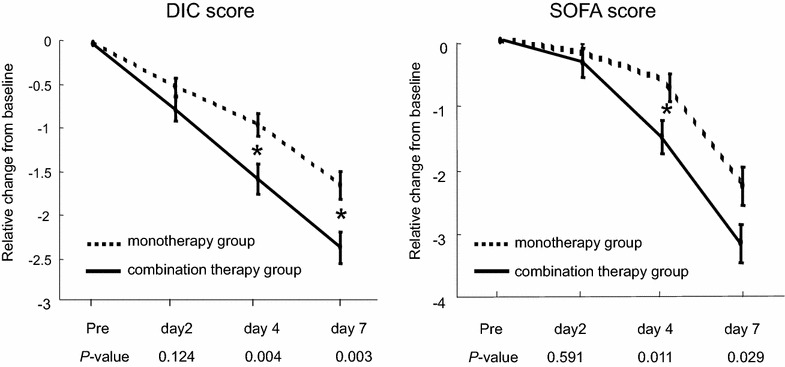
Changes in JAAM-DIC score and SOFA score in the propensity score-matched combination therapy and monotherapy groups. The JAAM-DIC scores were significantly lower in the combination therapy group than in the monotherapy group on Day 4 and Day 7 (*P* = 0.004, 0.003, respectively). The SOFA scores were significantly lower in the combination therapy group on Day 4 (*P* = 0.011) and Day 7 (*P* = 0.029), *DIC* disseminated intravascular coagulation, *SOFA* sequential organ failure assessment; *P < 0.017

The FDP level was significantly lower in the combination therapy group on Day 7 (*P* = 0.002). A significant difference in the PT ratio was observed on Day 7 between the groups (*P* = 0.014). The relative changes in JAAM-DIC score were significantly larger for the combination therapy group than for the monotherapy group on Day 4 and Day 7 (*P* = 0.004, 0.003, respectively). The relative changes in SOFA scores were significantly larger in the combination therapy group on Day 4 (*P* = 0.011) (Fig. 3).

### Bleeding events

Eighty-four cases presented with bleeding at the time of the diagnosis of DIC were not included among the bleeding events. Twenty cases which have no bleeding records before or after treatment were also excluded from the analysis. Bleeding events observed after diagnosis occurred in 4 out of 190 cases (2.11% [major: 1 case, 0.53%]) in the combination therapy group and in 5 out of 216 cases (2.31% [major: 3 case, 1.39%]) in the monotherapy group. The difference in the bleeding rate was not significant between the two groups (*P* = 1.000 [major: *P* = 0.626]). The details of the bleeding events are summarized in Table 4.

**Table 4:** Bleeding complications

No.	Treatment group	Bleeding site	Major/minor
Unmatched group			
1	Combination therapy	Mesenterium and intraperitoneal space	Major
2	Combination therapy	Abdominal wall, port site	Minor
3	Combination therapy	Intraperitoneal space, abdominal drain	Minor
4	Combination therapy	Urinary tract	Minor
5	Monotherapy	Intracranial space	Major
6	Monotherapy	Intraperitoneal space	Major
7	Monotherapy	Cervical spinal cord tumor	Major
8	Monotherapy	Urinary tract	Minor
9	Monotherapy	Nasal mucosa	Minor
Matched group			
5	Monotherapy	Intracranial space	Major
7	Monotherapy	Cervical spinal cord tumor	Major
9	Monotherapy	Nasal mucosa	Minor

## Discussion

Though this study was conducted to examine the effect of combination therapy, the comparison was performed between a combination therapy group and an antithrombin monotherapy group. Hence, the effect of recombinant thrombomodulin as an addition to antithrombin treatment was examined practically. However, since previous studies have demonstrated the possible efficacy of antithrombin substitution for sepsis-associated DIC [9, 10], we think that the results of the current study support the favorable effects of combination therapy. As for the effect of antithrombin substitution, a study using real-world data from a nationwide administrative database in Japan reported a beneficial effect [14]. A total of 9075 patients with severe pneumonia-associated DIC were categorized into an antithrombin group (2663 cases) and a control group (6412 cases). Propensity score matching created a matched cohort of 2194 pairs of patients with and without antithrombin treatment. The results demonstrated that standard antithrombin supplementation (1500 IU/day × 3 days) was associated with a 9.9% (95% CI 3.5**–**16.3%) reduction in the 28-day mortality rate (with antithrombin vs. without antithrombin: 40.6 vs. 44.2%). In addition, multiple logistic regression analyses showed an association between antithrombin use and the 28-day mortality rate. Similar results in peritonitis-associated DIC patients have also been reported [15]. Based on these reports, we think that the results of the current study suggested the additive effects of recombinant thrombomodulin to antithrombin therapy in patients with sepsis-associated DIC.

With respect to the effect of recombinant thrombomodulin, a phase 3 randomized controlled trial (RCT) comparing recombinant thrombomodulin and heparin in 234 patients with DIC associated with hematologic malignancy or infection was performed in Japan [16], and a subgroup analysis for infection-based DIC revealed that although the mortality difference was 10.2% (recombinant thrombomodulin: 21.4 vs. heparin: 31.6%), the difference was not statistically significant (95% CI − 9.1 to 29.4%) [17]. Since then, the effectiveness of this new agent has been repeatedly evaluated. For example, Yamakawa et al. [18] reported a trend toward favorable outcomes in their systematic review based on a meta-analysis. They collated data from 12 studies (838 patients from 3 RCTs and 571 patients from 9 observational studies) and reported that the relative risk of death was 0.81 (95% CI 0.62–1.06) in the RCTs and 0.59 (95% CI 0.45–0.77) in the observational studies. In contrast, Tagami et al. [19] performed propensity score and instrumental variable analyses using a Japanese nationwide administrative database (matched cohort of 1140 pairs) and reported that treatment with recombinant thrombomodulin did not reduce mortality among patients with pneumonia-associated DIC. More recently, Hagiwara et al. [20] performed an RCT at a single institute with 92 cases and reported an improved DIC resolution rate but almost identical mortality rates. The reason for these contradictory results has not yet been clarified; however, the severity of the subjects might affect the discrepancy. The beneficial effect of anticoagulants generally increases along with the severity of sepsis, and the reported effect was more evident if the study targeted severer cases [21, 22]. Yoshimura et al. [23] performed a post hoc analysis using data from a multicenter retrospective cohort study and reported that the administration of recombinant thrombomodulin was significantly associated with reduced mortality among patients with a high risk of death (APACHE II score: 24–29). Other than the above-mentioned studies, the largest RCT was conducted in 233 ICUs in 17 countries. A total of 750 patients with septic coagulopathy were randomized, and the results revealed a 3.8% reduction in the absolute risk of death (recombinant thrombomodulin group: 17.8% vs. placebo group: 21.6%, *P* = 0.273) [24]. This phase 2b study demonstrated a nonsignificant preferable effect of recombinant thrombomodulin. Regarding this study, one must keep in mind that not all patients had sepsis-associated DIC and the greatest benefit from the treatment was seen in patients with at least one organ system dysfunction and an PT international normalized ratio of greater than 1.4. In the current study, all the patients had DIC, the PT time was 1.61 ± 0.85 in the monotherapy group and 1.56 ± 0.54 in the combination therapy group, and the baseline SOFA scores were over 10 in both groups. Indeed, this was the first report to analyze matching data. As a result, a significant association between combination therapy and the 28-day mortality was recognized, and the mortality rate was significantly lower in the combination therapy group. In addition, both the JAAM-DIC score and the SOFA score were significantly lower in the combination therapy group than in the monotherapy group after the treatment (Days 4 and 7).

Regarding the safety features of recombinant thrombomodulin, a post-marketing surveillance of 2516 septic patients with DIC demonstrated that the frequency of critical bleeding was 2.6% [25], which did not differ from the results of the current study, suggesting that combination therapy might not increase the incidence of bleeding. However, since a significant number of patients were excluded from this analysis, this issue should be re-examined.

The theoretical rationale for combination therapy remains to be elucidated. However, a fundamental concept is that both antithrombin and thrombomodulin activities are significantly reduced and anticoagulatory function is disrupted during sepsis. Second, antithrombin and thrombomodulin–protein C are the two major anticoagulant systems and their mechanisms of action are independent. Third, both agents are expected to have anti-inflammatory actions [26, 27]. We think that the results obtained from the current study may support the above ideas. As for preclinical studies, we have examined the additive effects of combination therapy in a lipopolysaccharide-induced rat model of septic DIC. As a result, combination therapy attenuated organ damage and histologic changes and led to an improvement in survival [28, 29]. Additional studies are required to clarify the mechanism of action.

## Limitations

First, we only compared the effect between a combination therapy group and an antithrombin monotherapy group. To examine the true effect of the combination therapy, a combination therapy group, a monotherapy group, and a control group treated without anticoagulant are needed. Second, the median age of the patients was relatively higher and over 70-year-old, and thus, it might be difficult to generalize to the other countries. Finally, this was a retrospective observational study. Since the beneficial effect of combination therapy was hypothesized by the current study, a prospective randomized study is necessary as the next stage of inquiry.

## Conclusion

 The potential benefits of the co-administration of antithrombin and recombinant thrombomodulin were examined in a multi-institutional observation study. A propensity score-matched analysis demonstrated that the combination therapy was associated with a reduced mortality among patients with sepsis-induced DIC. Furthermore, the bleeding incidence seemed sufficiently low and the addition of recombinant thrombomodulin did not appear to increase the risk of bleeding.

## Additional file

**Additional file 1.** Japanese Association for Acute Medicine (JAAM) Disseminated Intravascular Coagulation (DIC) diagnostic criteria.

## Abbreviations

DIC: disseminated intravascular coagulation
OR: odds ratio
JAAM: Japanese Association for Acute Medicine
JMHW: Japanese Ministry of Health, Labour and Welfare
FDP: fibrinogen/fibrin degradation products
PT: prothrombin time
SIRS: systemic inflammatory response syndrome
SOFA: sequential organ failure assessment
VIF: variance inflation factor
IPW: inverse probability of treatment-weighted
SD: standard deviation
HR: hazard ratios
RCT: randomized controlled trial
