# Supplementary material for: Effects of combination therapy using antithrombin and thrombomodulin for sepsis-associated disseminated intravascular coagulation
Source: Ann Intensive Care. 2017 Nov 2;7:110. doi: 10.1186/s13613-017-0332-z (PMC5668219; doi:10.1186/s13613-017-0332-z)
Supplement: Supplementary file 1 — Additional file 1. Japanese Association for Acute Medicine (JAAM) Disseminated Intravascular Coagulation (DIC) diagnostic criteria. [file 13613_2017_332_MOESM1_ESM.docx]

**Supplement 1. Japanese Association for Acute Medicine (JAAM) Disseminated Intravascular Coagulation (DIC) diagnostic criteria**

|  | Score |
| --- | --- |
| Systemic inflammatory response syndrome criteria |  |
| ≥3 | 1 |
| 0–2 | 0 |
| Platelet count (×10^9^/L)  <80 or >50% decrease within 24 hours | 3 |
| ≥80 and <120, or 30% decrease within 24 hours  ≥120  Prothrombin time ratio  ≥1.2 | 1  0  1 |
| <1.2 | 0 |
| Fibrin/fibrinogen degradation products (FDP) (μg/mL)* |  |
| ≥25 | 3 |
| ≥10 and <25 | 1 |
| <10 | 0 |
| Diagnosis |  |
| ≥4 points | JAAM-DIC |

*: JAAM-DIC is also calculated by D-dimer.

The scoring for D-dimer is determined by using D-dimer/FDP conversion table

shown blow.

D-dimer/FDP conversion table*

|  | FDP 10ug/mL | FDP 25ug/mL |
| --- | --- | --- |
| Reagents/Kits | D-dimer (μg/mL) | D-dimer (μg/mL) |
| Sysmex Corporation | 5.4 | 13.2 |
| Nissui Pharmaceutical Corporation | 10.4 | 27.0 |
| BioView Corporation | 6.5 | 8.82 |
| LSI Medience Corporation | 6.63 | 16.31 |
| Roche Diagnostics Corporation | 4.1 | 10.1 |
| Sekisui Medical Corporation | 6.18 | 13.26 |
| Radiometer Corporation | 4.9 | 8.4 |
